# Normal versus Pathological Cardiac Fibroblast-Derived Extracellular Matrix Differentially Modulates Cardiosphere-Derived Cell Paracrine Properties and Commitment

**DOI:** 10.1155/2017/7396462

**Published:** 2017-06-27

**Authors:** Francesca Pagano, Francesco Angelini, Clotilde Castaldo, Vittorio Picchio, Elisa Messina, Sebastiano Sciarretta, Ciro Maiello, Giuseppe Biondi-Zoccai, Giacomo Frati, Franca di Meglio, Daria Nurzynska, Isotta Chimenti

**Affiliations:** ^1^Department of Medical Surgical Sciences and Biotechnologies, “La Sapienza” University of Rome, Rome, Italy; ^2^Department of Public Health, University of Naples “Federico II”, Naples, Italy; ^3^Department of Pediatrics and Childhood Neuropsychiatry, “Umberto I” Hospital, “La Sapienza” University of Rome, Rome, Italy; ^4^Department of Angiocardioneurology, IRCCS Neuromed, Pozzilli, Italy; ^5^Department of Cardiothoracic Sciences, Monaldi Hospital, Second University of Naples, Caserta, Italy

## Abstract

Human resident cardiac progenitor cells (CPCs) isolated as cardiosphere-derived cells (CDCs) are under clinical evaluation as a therapeutic product for cardiac regenerative medicine. Unfortunately, limited engraftment and differentiation potential of transplanted cells significantly hamper therapeutic success. Moreover, maladaptive remodelling of the extracellular matrix (ECM) during heart failure progression provides impaired biological and mechanical signals to cardiac cells, including CPCs. In this study, we aimed at investigating the differential effect on the phenotype of human CDCs of cardiac fibroblast-derived ECM substrates from healthy or diseased hearts, named, respectively, normal or pathological cardiogel (CG-N/P). After 7 days of culture, results show increased levels of cardiogenic gene expression (NKX2.5, CX43) on both decellularized cardiogels compared to control, while the proportion and staining patterns of GATA4, OCT4, NKX2.5, ACTA1, VIM, and CD90-positive CPCs were not affected, as assessed by immunofluorescence microscopy and flow cytometry analyses. Nonetheless, CDCs cultured on CG-N secreted significantly higher levels of osteopontin, FGF6, FGF7, NT-3, IGFBP4, and TIMP-2 compared to those cultured on CG-P, suggesting overall a reduced trophic and antiremodelling paracrine profile of CDCs when in contact with ECM from pathological cardiac fibroblasts. These results provide novel insights into the bidirectional interplay between cardiac ECM and CPCs, potentially affecting CPC biology and regenerative potential.

## 1. Introduction

Despite remarkable progress in early diagnosis and prevention, heart failure (HF) is still the leading cause of death in Western countries [1]. To date, heart transplantation could be considered the only effective therapeutic strategy for end-stage HF patients, albeit limited by organ availability and immunological issues. Accordingly, research has been focused on the development of alternative therapies able to repair a damaged heart and restore its function. Cardiac stem cell niches in postnatal hearts have been described in recent years [2, 3]. Resident cardiac progenitor cells (CPCs) can be isolated with several protocols [4] yielding mesenchymal-like cell populations sharing similar transcriptomic profiles [5]. Human CPCs can be isolated with clinically compliant protocols [6] and have been tested in few clinical trials as a promising tool for cardiac regenerative medicine [7, 8]. Unfortunately, despite the positive preclinical results [9, 10], regenerative medicine still cannot be considered a strong alternative to transplantation. It has been demonstrated that only 5–10% of the injected cells can be detected after 1 day from the procedure in the damaged myocardium, meaning that many cells are lost within few hours after injection [11, 12]. Furthermore, limited engraftment and proliferation and differentiation potential of the transplanted cells, together with the unsuitable ischemic microenvironment and the progressive myocardial maladaptive remodelling process, hamper the therapeutic outcome [13, 14]. Therefore, increasing the engraftment and regenerative potential of CPCs, as well as their antiremodelling capacities, for example, by means of tissue engineering approaches [15, 16] or pharmacological treatments [14, 17], would be beneficial.

In the heart, the extracellular matrix (ECM) mediates the connection among cardiomyocytes, cardiac fibroblasts (CFs), and blood vessels, granting optimal mechanical features and sustaining cardiac functions [18, 19]. CFs are one of the most abundant resident noncardiac cell subpopulations in the heart. These cells produce and secrete ECM components (e.g., collagens, fibronectin) and, at the same time, maintain its homeostasis, through the production of matrix metalloproteinases (MMPs) and tissue inhibitors of metalloproteinases (TIMPs) [20]. It is well known that an imbalanced deposition of ECM components and maladaptive ECM remodelling are detrimental mechanisms contributing to the progression of HF [21–24]. This effect is due to both impaired biological and mechanical stimuli on all cardiac cells, including CPCs. It has been recently described that cardiac fibroblast-derived ECM from normal or pathological hearts can affect in many ways proliferation, migration, and resistance to apoptosis of CPCs [25], but its effects on cardiovascular commitment, phenotype, and paracrine properties of CPCs have not been elucidated yet. The aim of the present study is to investigate in vitro the molecular and functional effects elicited on CPC phenotype when cultured on cardiac fibroblast-derived ECM substrates, in order to better understand the interactions between ECM components and a suitable cell product candidate for heart regenerative therapy, as well as to improve experimental protocols.

## 2. Materials and Methods

### 2.1. Cardiac Fibroblast Isolation and Cardiogel Deposition

Cardiac samples of the right atrial appendage of human hearts were obtained from both donor (*n* = 9, mean age 50.4 ± 4.1 years) and recipient (*n* = 9, mean age 55.8 ± 3.1 years) of heart transplantation. Patients (or legally legitimate relatives/guardians) provided written informed consent, and specimens were collected without patient identifiers following protocols approved by Monaldi Hospital, and in conformity with the principles outlined in the Declaration of Helsinki. Cardiac extracellular matrix synthesized and deposited in vitro was prepared as previously described [26]. Briefly, samples were dissected, minced, and enzymatically digested by incubation in 0.25% trypsin and 0.1% (*w*/*v*) collagenase II (both from Sigma-Aldrich, St. Louis, MO, USA) for 30 minutes at 37°C. Digestion ceased when double volume of HBSS (Sigma-Aldrich) supplemented with 10% fetal bovine serum (FBS) (Sigma-Aldrich) was added. Further mechanical disaggregation was achieved by pipetting; hence, tissue debris and cardiomyocytes were removed by sequential centrifugation at 100 rcf for 2 minutes, passage through a 20 *μ*m cell strainer, and centrifugation at 400 rcf for 5 minutes. Isolated fibroblasts, either from normal (healthy) or from pathological cardiac tissue (from donors or recipients, resp.), were cultured in DMEM (Sigma-Aldrich) supplemented with 10% FBS (Sigma-Aldrich) and maintained in a confluent state for 21 days, to allow extracellular matrix synthesis and deposition. Next, fibroblasts were removed with a nonenzymatic method. Fibroblasts were removed by incubation for 1-2 minutes with a solution of 0.25% Triton X-100 and 10 mM NH_4_OH in PBS. Culture dishes coated with fibroblast-derived matrix, here named cardiogel, were washed and used for CPC culture.

### 2.2. Cardiosphere-Derived Cell Culture

CPCs were isolated as cardiosphere-derived cells (CDCs), as previously described [27], which are a clinically relevant therapeutic cell population [28] of nonhematopoietic stromal cells containing CPCs [29–31]. CDCs were derived from right atrial appendage biopsies obtained from three donor patients undergoing elective cardiac surgery during clinically indicated procedures, after informed consent, in an institutional review board-approved protocol at the “Umberto I” Hospital, “La Sapienza” University of Rome. CDCs were seeded at a density of 1.25 × 10^4^ cells/cm^2^ in Petri dishes previously coated with normal or pathological cardiogel (CG-N and CG-P, resp.). Cells were then cultured for 7 days in complete explant media (CEM) [Iscove's modified Dulbecco's medium (IMDM) (Sigma-Aldrich) supplemented with 3% FBS (Sigma-Aldrich), 1% penicillin-streptomycin (Sigma-Aldrich), 1% L-glutamine (Lonza, Basel, Switzerland), and 0.1 mM 2-mercaptoethanol (Gibco, Thermo Fisher Scientific, Waltham, MA, USA)], using cells grown on fibronectin-coated plates at the same density as the control group.

### 2.3. Immunostaining and Fluorescence Microscopy Analyses

After 1 week of culture in 3% FBS-CEM on each cardiogel and on fibronectin-coated control plates, CDCs were fixed for 10 minutes with 4% paraformaldehyde at 4°C. For immunofluorescence, cells were permeabilized with 0.1% Triton X-100 (Sigma-Aldrich) in PBS with 1% BSA. Nonspecific antibody binding sites were blocked with 10% goat serum (Sigma-Aldrich) before overnight incubation at 4°C with primary antibodies: ACTA1, GATA-4, OCT-4, NKX2-5 (all Abcam, Cambridge, UK), CX43, KDR (all Millipore, MA, USA), and vimentin (Santa Cruz Biotechnology, Dallas, TX, USA). After thorough washing, slides were incubated for 2 hours at room temperature with the appropriate Alexa-conjugated secondary antibodies (Invitrogen, Carlsbad, CA, USA) and DAPI nuclear dye (Invitrogen). Slides were mounted in 70% PBS-glycerol. Imaging was performed on a Nikon Eclipse Ni microscope equipped with VICO system and NIS-Elements AR 4.30.02 software (Nikon Corporation, Tokyo, Japan).

### 2.4. Flow Cytometry Analysis

Cells were grown on CG-N and CG-P, and the percentage of cells expressing CD90 was assessed by flow cytometry. Briefly, the cells were harvested with gentle trypsin-EDTA treatment and stained with CD90-FITC (Dianova, Hamburg, DE) antibody diluted to 1 : 100 in PBS-2% FBS. Samples were analyzed with a FACS Aria II cytometer (BD Biosciences, San Jose, CA, USA) using Diva software (version 6.1.1; BD Biosciences). Data was analyzed with FlowJo software (version 2.5.1; Turku Centre for Biotechnologies, Turku, Finland, http://www.btk.fi).

### 2.5. RNA Extraction and Real-Time PCR

Total RNA was extracted using the miRNeasy Micro Kit (Qiagen, Hilden, DE) and quantified using a spectrophotometer. cDNA was synthesized using 0.5 *μ*g RNA, with the High-Capacity cDNA Reverse Transcription Kit (Life Technologies, Thermo Fisher Scientific, Waltham, MA, USA). Real-time qPCR was performed to assess gene expression, using Power SYBR Green PCR Master Mix (Life Technologies, Thermo Fisher Scientific) and standard thermocycling conditions according to the manufacturer's protocol. The relative ratio for each substrate versus culture on fibronectin was calculated using the comparative Ct method (2^−ΔΔCt^) for each patient sample. The set of genes analyzed and the primers sequences are listed in Table 1. GAPDH was selected according to the NormFinder software, as a housekeeping gene.

### 2.6. Conditioned Medium Screening

After 5 days of culture, media were changed for the last 24 hours of culture to be collected and analyzed for the presence of cytokines. All CDC cultures were in equivalent cell number/volume of media ratio. Media were centrifuged at 2000 rcf for 5 minutes and then stored at −80°C until analysis. Media were analyzed by a membrane-based ELISA (RayBio® Human Cytokine Antibody Array 5; RayBiotech, Norcross, GA, USA), according to the manufacturer's instructions. Densitometric analysis was performed by ImageJ software, and data is presented as optical density values normalized to the assay's internal positive control.

### 2.7. Statistical Analysis

All results are presented as mean value ± standard error of the mean, unless specified. Significance of difference between any two groups was determined by two-sided Student's *t*-test, and a final value of *p* < 0.05 was considered significant.

## 3. Results

**Figure 1:**
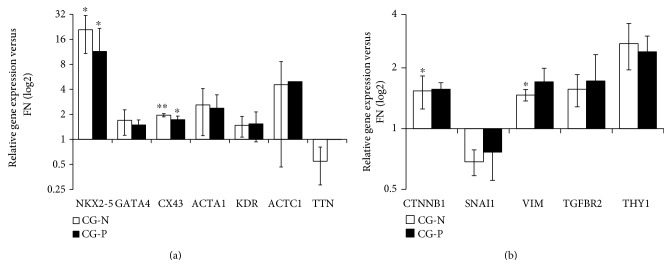
Relative gene expression levels of CDCs grown on normal (CG-N) and pathological cardiogel (CG-P). Bar graphs showing gene expression change in a panel of cardiac differentiation and commitment (a) and mesenchymal (b) genes. Both CG-N and CG-P CDCs displayed significantly higher expression levels of the cardiac-specific genes NKX2-5 and CX43 (a) compared to CDCs grown on fibronectin used as the control. Only CTNNB (beta catenin) and VIM (b) were significantly upregulated in CG-N and CG-P CDCs, respectively, compared to the control (*n* = 3). CG-N: normal cardiogel; CG-P: pathological cardiogel; ACTC1: cardiac muscle actin alpha; TTN: titin. ^∗∗^*p* < 0.01, ^∗^*p* < 0.05.

**Figure 2:**
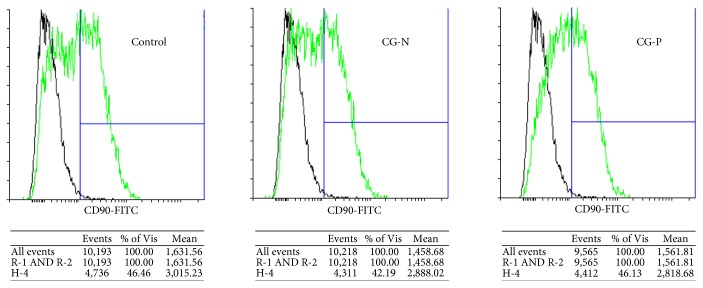
CD90-positive CDC immunophenotype on different cardiogel substrates. Representative histogram of flow cytometry analysis. The immunophenotypes of CG-N and CG-P CDCs were not significantly different concerning the abundance of the CD90^+^ subpopulation, as shown by the representative flow cytometry histograms. CG-N: normal cardiogel; CG-P: pathological cardiogel.

**Figure 3:**
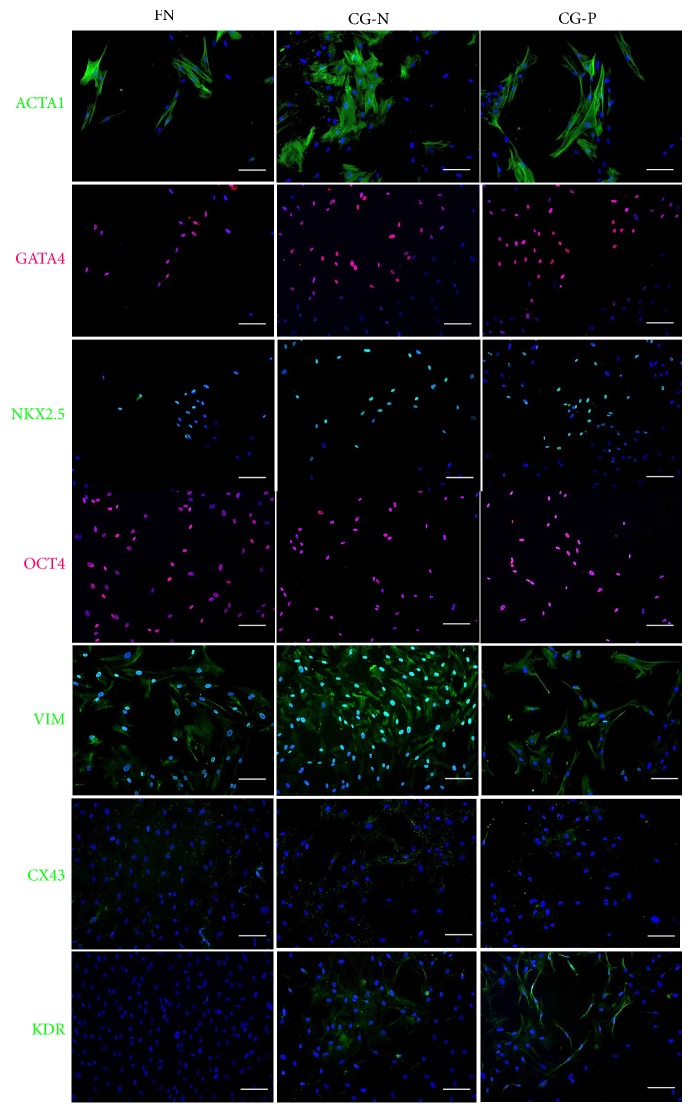
Immunofluorescence phenotype of CDCs from normal and pathological cardiogels. Representative CDC immunofluorescence images showing no differences in the distribution of the proteins ACTA1, GATA4, NKX2.5, OCT4, and VIM between cells grown on CG-N and CG-P. The protein expression of Cx43 and KDR increases in CDCs cultured on cardiogel. Scale bars = 100 *μ*m. FN: fibronectin; CG-N: normal cardiogel; CG-P: pathological cardiogel.

We obtained decellularized matrix, named cardiogel (CG), from confluent cultures of endogenous CFs, isolated from biopsies of normal (CG-N) or pathological (CG-P) heart tissue, as previously described [25]. CPCs were isolated from cardiospheres as CDCs, as an established spheroid culture system to isolate progenitor cells from solid tissues [27, 32]. CDCs were plated and grown for 7 days on the two different substrates, CG-N and CG-P, and on standard fibronectin-coated dishes as the control. We assessed the expression levels of three panels of marker genes related to CPC phenotype, epithelial-to-mesenchymal transition, and cardiovascular commitment and differentiation. We observed a significant increase in the expression of the cardiac-specific transcription factor NK2 homeobox 5 (NKX2-5) in both CGs and control (Figure 1(a)), suggesting that the cells are subjected to more physiological and cardiogenic stimuli by the substrates. We observed also a significant upregulation of the connexin 43 gene expression (CX43) in both cardiogels compared to FN-coated controls (Figure 1(a)), suggesting a cardiac differentiation-supportive signal from both CGs compared to standard culture conditions. The results for all other cardiovascular genes analyzed showed trends without reaching statistically significant changes. Concerning EMT-related genes, we could observe a slight modulation among samples of *β*-catenin (CTNNB1) and vimentin (VIM) expression (Figure 1(b)). We evaluated the percentage of CD90^+^ cells by flow cytometry, since this parameter has been linked to lower performance of the CDC cellular product in cell therapy approaches [33, 34], and found that the CD90^+^ subpopulation was comparable among samples from both cardiogels (Figure 2). We also analyzed in the same samples the proportion of CD117^+^ cells, as a proposed marker for CPCs [35], and it was unaffected (data not shown). Next, we investigated by immunofluorescence staining whether the protein abundance or distribution of markers related to stemness and cardiac commitment could be modulated by the different substrates (Figure 3). The percentage of cells positive for the transcription factors POU class 5 homeobox 1 (OCT-4), NKX2-5, and GATA-binding protein 4 (GATA4) was not affected by culture substrates (Supplementary Figure 1 available online at https://doi.org/10.1155/2017/7396462), as well as the proportion and morphology of VIM-positive and *α*-smooth muscle actin (ACTA1)-positive cells (Figure 3). Instead, we observed a staining pattern of connexin 43 and vascular endothelial growth factor receptor (KDR), showing an increase in these protein expression in cells cultured on cardiogels (Figure 3).

**Figure 4:**
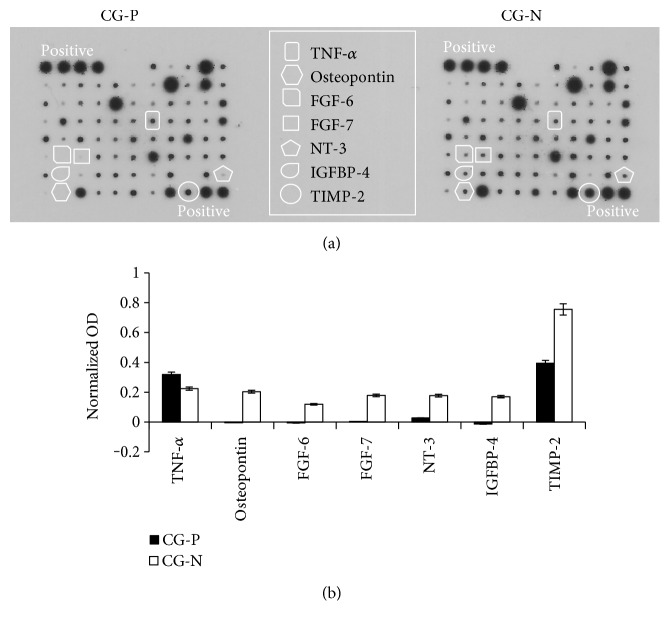
Screening of conditioned media from CG-N and CG-P CDCs for paracrine molecules. (a) Representative blots from protein arrays of CDC-conditioned media. A selection of growth factors, chemokines, and cytokines of interest is highlighted in the panel. Differential analysis of conditioned media from CG-N and CG-P CDCs revealed a distinctive modulation in the secretion profile of a subset of cytokines, as plotted in (b). OD: optical density; CG-N: normal cardiogel; CG-P: pathological cardiogel.

CDCs have been shown to exert paracrine beneficial effects [36–38]; therefore, we investigated whether culture on CGs could differentially influence their secretion profile. We screened 24 hour-conditioned media by protein arrays and detected the modulation of several humoral factors on the different substrates (Figure 4(a)). Most of the cytokines were not differentially secreted in conditioned media collected from CG-N and CG-P cultures. Nonetheless, osteopontin, fibroblast growth factor 6 (FGF6), fibroblast growth factor 7 (FGF7), neurotrophin 3 (NT-3), insulin-like growth factor-binding protein 4 (IGFBP4), and tissue inhibitor of metalloproteinases 2 (TIMP2) levels were significantly upregulated in CG-N-conditioned media compared to CG-P-conditioned media (Figure 4(b)).

## 4. Discussion

One major topic of interest in the field of CPC biology is how their differentiation potential may be influenced by changes in ECM features during both normal aging and HF remodelling. These two conditions may have additive effects on the reduction in the stemness potential of resident CPCs [39], which may conversely affect ECM homeostasis and remodelling. Thus, it is important to understand the mechanisms of the bidirectional relationship between ECM and CPCs, because the presence of pathological ECM in a site of cell therapy delivery/engraftment may hamper the regenerative potential of resident progenitors, as well as that of exogenous transplanted CPCs, through detrimental biomechanical signals [19, 40], altering their cardiogenic/fibrotic balance [41]. Consistently, despite the abundance and therapeutic phenotype of CPCs isolated from advanced HF patients [42], the differentiation process of resident CPCs has been demonstrated to slow down in pathological conditions, with higher accumulation of progenitors versus precursors [43]. In the present study, we investigate the effects on CDC phenotype (which is a clinically relevant cell population containing CPCs [28]) elicited by an ex vivo cardiac fibroblast-derived decellularized ECM substrate, named cardiogel, laid by CFs isolated either from normal (healthy) or from pathological heart tissue. This substrate has been previously characterized; it is made of several different endogenous cardiac proteins, with a higher protein content in laminin, tenascin, and collagen I when produced by pathological CFs, and induces a higher proliferation of primitive CD117^+^ cardiac cells [25]. Here, we report that the phenotype of CPCs is partially affected by culture on cardiogels; specifically, we observed upregulated gene expression of NKX2-5 and CX43, similar on both substrates as compared to standard culture conditions. However, this transcriptional modulation was confirmed by immunostaining for CX43 protein only. In addition, despite KDR gene expression change in CGs does not reach statistical significance, the protein level is increased in cells grown on both cardiogel substrates. These results suggest a partial commitment effect of the cardiogels on CDCs. The analysis of indicators of mesenchymal fibroblast-like phenotype revealed no significant modulation at the transcriptional level, except for a slight increase in vimentin gene expression in CG-P, although not mirrored by evident modulation in the corresponding protein level or distribution. Also, the CD90^+^ subpopulation proportion, the only immunophenotypical trait reported so far to significantly impair the therapeutic potential of CDCs [33] and to be associated with features of a fibrotic-prone phenotype [17, 34], was not affected by the different substrates. Overall, these data suggest that the cardiogel substrates tested do not change, at least in the short term, the phenotypic traits within the CDC pool or their ability to undergo type 2 EMT [44] towards a fibrotic phenotype.

CPCs exert therapeutic effects through both direct and indirect mechanisms, such as cardiovascular differentiation and paracrine effects [36–38]. Interestingly, culture on CG-N was associated with significant upregulation of some secreted cytokines of interest compared to that on CG-P, all of which have been shown to exert positive effects on cardiac cell types in different contexts. The release of TNF-*α* by CPCs was the only one reduced on CG-N compared to CG-P, and since it is a main inflammatory mediator of ischemic injury [45] and a potential target of beneficial cell therapy mechanisms [46], this reduction is consistently associated with the more physiological substrate (CG-N). Concerning the cytokines secreted at higher levels in CG-N, the FGF family in mammals comprises many different proteins, where FGF2, FGF16, FGF21, and FGF23 are the main forms known to regulate cardiac physiology and pathophysiology [47]. Despite the fundamental homeostatic roles of FGF family isoforms, such as FGF2, in the heart [48, 49], to the best of our knowledge, many other members of the FGF family, including FGF6 and FGF7, have not been directly associated with any cardiac condition. Nonetheless, it has been reported that FGF6 has a dual function in muscle regeneration, stimulating myoblast proliferation/migration and muscle differentiation/hypertrophy in a dose-dependent way. Moreover, FGF6 has been suggested to play a role in the maintenance of a progenitor cell pool in the skeletal muscle [50]. We might speculate that similar mechanisms may be active also in the heart. Osteopontin, instead, is known to be upregulated in multiple tissues, including those in the heart, in response to injury and inflammation [51], and its balanced release has been described to sustain impaired angiogenesis during tissue repair after infarction [52]. NT-3 downregulation, instead, is associated with impaired sympathetic function in the heart during the progression of heart failure [53], suggesting that CPCs grown on CG-N may be more prone to counteract this detrimental mechanism. Moreover, TIMP-2 release was also increased on CG-N versus CG-P. TIMP-2 is an MMP inhibitor acting as an antiremodelling secreted protein. Increased MMP activity has been associated with HF progression [54], and TIMPs are currently proposed as novel biomarkers of positive prognosis [55], thus supporting the potential anti-ECM remodelling effect of CPCs when in contact with CG-N, that is, ECM laid by fibroblasts from healthy cardiac tissue. IGFBP4 is an IGF1-binding protein regulating the bioavailability of IGF1, which is a pathway previously described to be active in CPCs [36], and highly regulated particularly in 3D spheroid culture conditions [56]. It is noteworthy that IGF1 and IGFBP4 expression has been reported to be upregulated during cardiac benign reverse remodelling processes [57], consistently with the overall antiremodelling CPC profile on CG-N supported by all the above-mentioned secreted factors. A clinically relevant correlation has been recently reported in humans between medical parameters associated, among others, with antifibrotic properties of resident CPCs and the reversal of maladaptive cardiac remodelling processes [17, 58, 59]. This further strengthens the model of bidirectional positive reinforcement between antiremodelling features of resident CPCs and beneficial ECM properties in pathological cardiac conditions. Further studies will be needed to assess how CG-N and CG-P may affect the biology of other cardiac cell types.

In conclusion, our results show that a human cardiac fibroblast-derived ECM substrate from HF tissue, that is, CG-P, does not directly affect short-term the phenotype of resident CPCs but nonetheless is associated with a more profibrotic and proremodelling paracrine profile of CPCs. These observations provide important novel insights into (1) the interplay between CPC and cardiac ECM; (2) how HF progression may affect the resident CPC pool through changes in ECM properties, and vice versa; and (3) how the remodelled ECM microenvironment of recipient hearts may negatively affect the paracrine profile of engrafted exogenous CPCs in cardiac cell therapy approaches.

## Supplementary Material

Supplementary figure 1: Quantification of immunofluorescence staining. Bargraph shows the percentage of cells positive for GATA4, NKX2-5 and OCT4 as per immunostatining shown in Figure 3.

## Figures and Tables

**Table 1:** Primers used for qPCR analyses.

GAPDH fw	ACAGTCAGCCGCATCTTC
GAPDH rv	GCCCAATACGACCAAATCC
Nkx2.5 fw	GGTGGAGCTGGAGAAGACAGA
Nkx2.5 rv	CGCCGCTCCAGTTCATAG
GATA-4 fw	GTTTTTTCCCCTTTGATTTTTGATC
GATA-4 rv	AACGACGGCAACAACGATAAT
Cx43 Fw	AGGAGTTCAATCACTTGGCG
Cx43 Rv	GAGTTTGCCTAAGGCGCTC
Thy-1 fw	CAGCGGAAGACCCCAGT
Thy-1 rv	CGTTAGGCTGGTCACCTTCT
ACTC1 fw	GTACCCTGGTATTGCTGATCG
ACTC1 rv	CCTCATCGTACTCTTGCTTGCT
TTN fw	CCTTGCCTGACACACCAGAT
TTN rv	GGTGCTGGTACTCTTGCTGT
CTNNB1 fw	AGGTCTGAGGAGCAGCTTCA
CTNNB1 rv	ATTGTCCACGCTGGATTTTC
Snai1 fw	CTTCTCTAGGCCCTGGCTG
Snai1 rv	CATCTGAGTGGGTCTGGAGG
TGFBR2 fw	CTGCACATCGTCCTGTGG
TGFBR2 rv	GGAAACTTGACTGCACCGTT
Vim rv	ACCCACTCAAAAAGGACACTTC
Vim fw	GGTCATCGTGATGCTGAGAA
ACTA1fw	ATGAAGATCCTGACTGAGCG
ACTA1 rv	GCAGTGGCCATCTCATTTTC
KDR fw	AAAGGGTGGAGGTGACTGAG
KDR rv	CGGTAGAAGCACTTGTAGGC
