# Supplementary material for: Normal versus Pathological Cardiac Fibroblast-Derived Extracellular Matrix Differentially Modulates Cardiosphere-Derived Cell Paracrine Properties and Commitment
Source: Stem Cells Int. 2017 Jun 27;2017:7396462. doi: 10.1155/2017/7396462 (PMC5504962; doi:10.1155/2017/7396462)
Supplement: Supplementary file 1 — Supplementary figure 1: Quantification of immunofluorescence staining. Bargraph shows the percentage of cells positive for GATA4, NKX2-5 and OCT4 as per immunostatining shown in Figure 3. [file 7396462.f1.pdf]

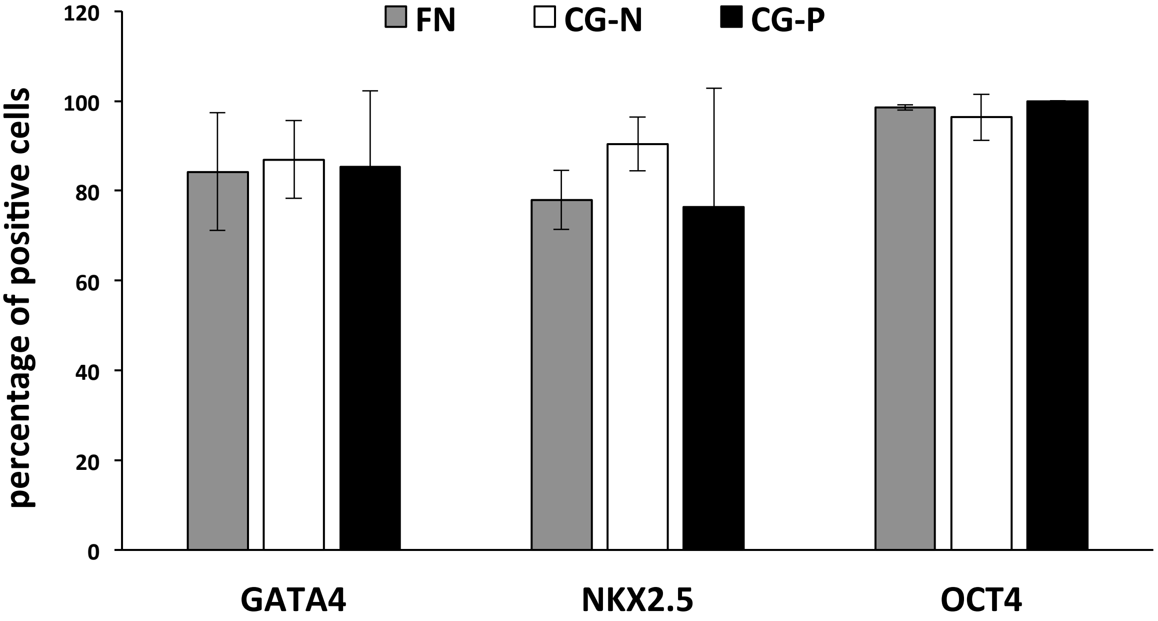

Supplementary figure 1: Quantification of immunofluorescence staining. Bargraph shows the percentage of cells positive for GATA4, NKX2-5 and OCT4 as per immunostatining shown in figure 3.
